# Temporal Trends in Surgery and Hospitalization Rates for Crohn’s Disease in Brazil: A Population-Based Study

**DOI:** 10.1093/crocol/otae082

**Published:** 2025-01-09

**Authors:** João Augusto dos Reis Guerra, Daniela Oliveira Magro, Claudio Saddy Rodrigues Coy, Douglas A Valverde, Emilia Sousa de Oliveira, Abel Botelho Quaresma, Paulo Gustavo Kotze

**Affiliations:** Colorectal Surgery Unit, Catholic University of Paraná, IBD Outpatient Clinics, Curitiba, Brazil; Colorectal Surgery Unit, University of Campinas UNICAMP, Campinas, Brazil; Colorectal Surgery Unit, University of Campinas UNICAMP, Campinas, Brazil; Techtrials Healthcare, Data Science, Vinhedo, Brazil; Department of Internal Medicine, University of Campinas UNICAMP, Campinas, Brazil; Health Sciences Postgraduate Program, Pontifícia Universidade Católica do Paraná (PUCPR), Curitiba, Brazil; IBD Outpatient Clinic, Colorectal Surgery Unit, Universidade do Oeste de Santa Catarina, UNOESC, Brazil; Colorectal Surgery Unit, Catholic University of Paraná, IBD Outpatient Clinics, Curitiba, Brazil; Health Sciences Postgraduate Program, Pontifícia Universidade Católica do Paraná (PUCPR), Curitiba, Brazil

**Keywords:** Crohn’s disease, surgery, hospitalizations, biological therapy, epidemiology

## Abstract

**Introduction:**

Biological therapy has transformed the natural course of inflammatory bowel disease, but there are still controversies regarding its potential to reduce surgical rates for Crohn’s disease (CD). This study, conducted with the support of the Brazilian National Healthcare System, aimed to analyze temporal trends in surgery and hospitalization rates among patients with CD and to correlate these data with the dispensing of azathioprine (AZA), infliximab (IFX), and adalimumab (ADA).

**Methodology:**

This retrospective observational study used data from the National Public Healthcare Department of Informatics through the TT Disease Explorer^®^ platform from 2012 to 2022. Demographic data, medications used, and the prevalence of surgical procedures and hospitalizations associated with the International Classification of Diseases codes for CD were analyzed. Annual average percent changes (AAPCs) were calculated to assess temporal trends.

**Results:**

Between 2012 and 2022, there was a significant increase of 288.07% in the diagnoses of CD, rising from 27 551 to 106 917 cases. Concurrently, there was an increase in the absolute number of patients treated with AZA, IFX, and ADA, with increasing rates of 65.79%, 251.09%, and 242.48%, respectively. However, the proportion of patients receiving AZA per CD patients decreased by 57.28%, from 44.79% to 19.13% (AAPC = −7.94%, 95% CI, −8.05 to −7.83; *P* < .001). The use of IFX remained relatively stable, with a slight change from 13.82% to 12.50% (AAPC = 0.01%, 95% CI, −0.20 to 0.22; *P* = .935), while the use of ADA decreased by 11.75%, from 11.65% to 10.28% (AAPC = −1.74%, 95% CI, −2.48 to −1.82; *P* < .001). The absolute number of hospitalizations related to CD increased by 57.71%. Despite the rise in the number of cases and the greater availability of biological treatments, the proportion of hospitalized patients decreased by 59.29%, from 6.19% to 2.52% (AAPC = −7.04%, 95% CI, −7.42 to −6.66; *P* < .001). Similarly, the proportion of surgical procedures relative to the total number of cases decreased by 55.08%, from 1.09% to 0.49% (AAPC = −5.73%, 95% CI, −6.68 to −4.77; *P* < .001).

**Conclusions:**

Despite the cumulative increase in the prevalence of CD cases in the country and the absolute increase in the dispensing of biologics, the proportion of hospitalizations and surgical procedures among CD patients treated in the public health system in Brazil decreased.

## Graphical Abstract

**Graphical Abstract:**
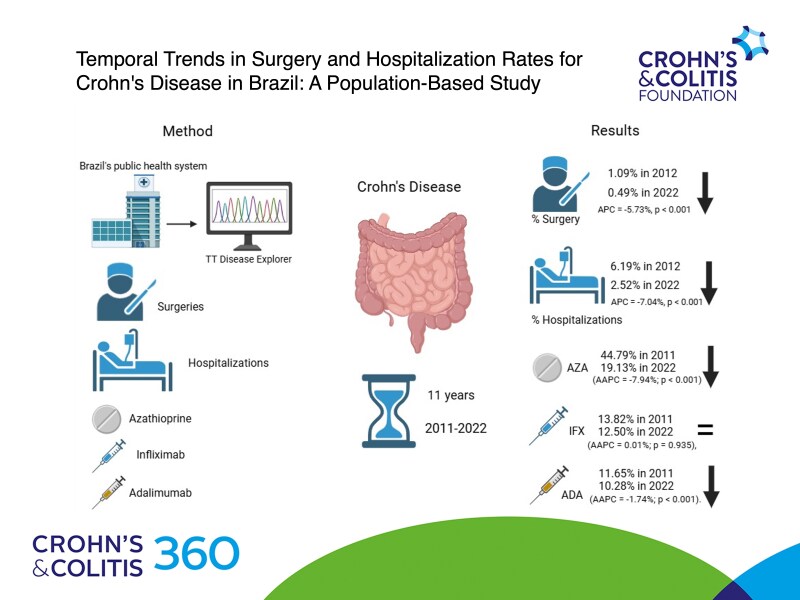

## Introduction

Crohn’s disease (CD) is a chronic, idiopathic inflammatory disorder of the gastrointestinal tract characterized by periods of activity and remission.^1^ The diagnosis can occur at any age but is most common among adolescents and young adults.^2^ Disease activity, access to healthcare services, and the availability of medications influence treatment outcomes, which may require multiple hospitalizations and surgical interventions.^3^ Within the first 5 years after diagnosis, approximately 28% of CD patients will undergo abdominal surgery, and within 10 years, this number rises to 39.5%.^4^

The advent of biologics has revolutionized the treatment of CD, leading to early symptom control, reduced need for hospitalizations and surgical procedures, and the possibility of complete mucosal healing.^5^ In the last 20 years, various protocols for monitoring and managing CD have been developed.^6^ This has allowed for a transition from hospital-based management to outpatient care for patients with moderate to severe CD, resulting in lower rates of hospitalizations and major abdominal surgeries.^7^

The progression of CD can be stratified into 4 epidemiological stages: the emergence of new cases, acceleration of incidence, worsening of prevalence, and prevalence equilibrium.^8^ Western countries are currently in worsening prevalence, where incidence rates are stable or declining. Still, prevalence rates are high, with 0.3% of the population affected in Europe and the United States and approximately 0.7% in Canada.^9,10^

In Latin America, there are few epidemiological studies on CD. Conducting these studies presents multiple challenges, including the region’s geographic, social, and economic diversity and the lack of a comprehensive, organized, and reliable database.^11^ A population-based study in Brazil, published in 2022, identified an incidence of CD at 2.68 new cases per 100 000 inhabitants and a prevalence of 33.68 cases per 100 000 inhabitants.^12^ These numbers are alarming, as the prevalence rates were significantly higher than those observed in previous studies.^13,14^

In newly industrialized countries like Brazil, assessing the impact of current medical options for CD on hospitalization and surgery rates can be challenging due to the limited number of studies addressing this topic in the national context. This study aims to describe the temporal trends of hospitalization and abdominal surgery rates related to CD and to evaluate their possible correlations with the dispensing of azathioprine (AZA), infliximab (IFX), and adalimumab (ADA) in Brazil, using public healthcare datasets.

## Methods

This retrospective, population-based study included all patients diagnosed with CD treated within Brazil’s public healthcare system (*Sistema Único de Saúde*) from January 2012 to December 2022. The study encompassed consultations, complementary tests, various forms of treatment (medical or surgical), and hospital admissions associated with the diagnosis of CD.

The public healthcare department of informatics (DATASUS) provides an open-access, population-based health and disease registry containing information from the public healthcare system on medical procedures, hospital admissions and discharges, mortality, and demographic variables, covering the entire national population.^15^ All data are anonymized and do not allow for the identification of individual subjects. Population data for prevalence calculation were extracted from the Brazilian Institute of Geography and Statisticswebsite.^16^

For the diagnosis of CD, the study used the codes K50, K50.0, K50.1, K50.8, and K50.9 from the 10th edition of the International Classification of Diseases and Related Health Problems (ICD-10). For pharmacological treatment, CD patients were classified into 3 groups based on their use of AZA, ADA, and IFX, as recorded by dispensing of these drugs in the database.

Data were collected from DATASUS using the TT Disease Explorer^®^ platform, developed by Techtrials (Techtrials Healthcare Data Science), which continuously collects epidemiological data, as well as diagnostic and therapeutic interventions. The platform allows for analyzing the number of hospitalizations and surgical procedures performed on CD patients. The database is automatically updated and does not permit duplication of patients, as each patient has a unique identification number. The platform identifies and prevents duplicates if the same patient undergoes procedures in different parts of the country. Although the platform contains data from 2008, we opted to use data from 2012 onwards, as these are more recent and standardized. The data were extracted over a total period of 11 fiscal years.

CD patients who underwent surgical procedures were classified into 2 groups: those who had abdominal surgeries and those who had anorectal procedures. Codes from the specific Procedure, Medication, Orthotics, and Prosthetics Management System (SIGTAP) were used to analyze surgical procedures.^17^ The abdominal surgery group included the most used procedures for the surgical management of the disease, such as abdominoperineal resection of the rectum, partial colectomy, total colectomy, laparoscopic colectomy, colorrhaphy, colostomy, enterectomy, enteroanastomosis, enterorrhaphy and/or enterectomy, closure of enterostomy, closure of colonic fistula, jejunostomy and/or ileostomy, anterior resection, and perineal rectosigmoidectomy. The anorectal surgery group included procedures for digital and/or instrumental dilation of the anus and/or rectum, drainage of anorectal abscess, drainage of ischiorectal abscess, excision of anorectal lesion and/or tumor, and anal fistulotomy. For the analysis of hospitalizations, patients diagnosed with CD with the same ICD codes related to CD were considered as the primary cause of admission in the Hospitalization Authorization.

Data were analyzed descriptively through frequency distribution. For trend analyses, annual percent changes and average annual percent changes (AAPCs), with 95% confidence intervals, were calculated using R and RStudio software, version 4.3.1. The local Research Ethics Committee (CEP-UNOESC HUST) approved the study protocol, under reference number 3.002.676.

## Results

Between 2012 and 2022, the number of unique patients diagnosed with CD increased by 288.07% (from 27 551 to 106 917), with 55.57% of patients being female. This growth raised the nationwide prevalence from 14.20 to 52.64 cases per 100 000 inhabitants.

**Figure 1.**
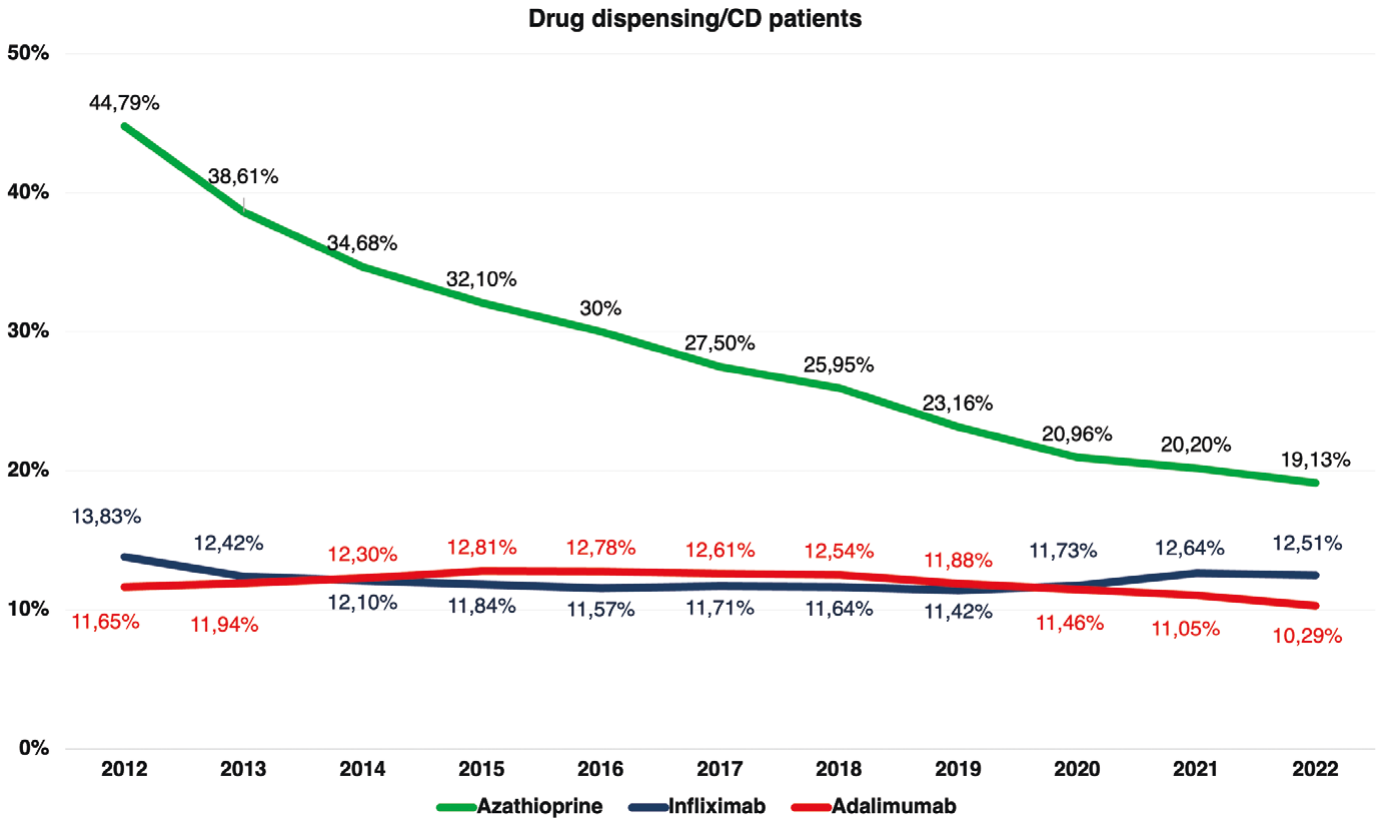
Proportion of patients by therapy used in Brazil’s Unified Health System from 2012 to 2022.

Regarding the type of therapy, between 2012 and 2022, there was an increase in the absolute number of patients receiving AZA, IFX, and ADA by 65.79%, 251.09%, and 242.48%, respectively. However, the proportion of patients (having all CD patients as a denominator) receiving AZA decreased by 57.28%, from 44.79% to 19.13% (AAPC = –7.94%, 95% CI, –8.05 to –7.83; *P* < .001). The proportion of patients receiving IFX remained stable (from 13.82% in 2012 to 12.50% in 2022, AAPC = 0.01%, 95% CI, –0.20 to 0.22; *P* = .935). Similarly, the proportion of patients receiving ADA showed a modest decrease of 11.75%, from 11.65% to 10.28% (AAPC = –1.74%, 95% CI, –2.48 to –1.82; *P* < .001). These data are illustrated in Figure 1.

**Figure 2.**
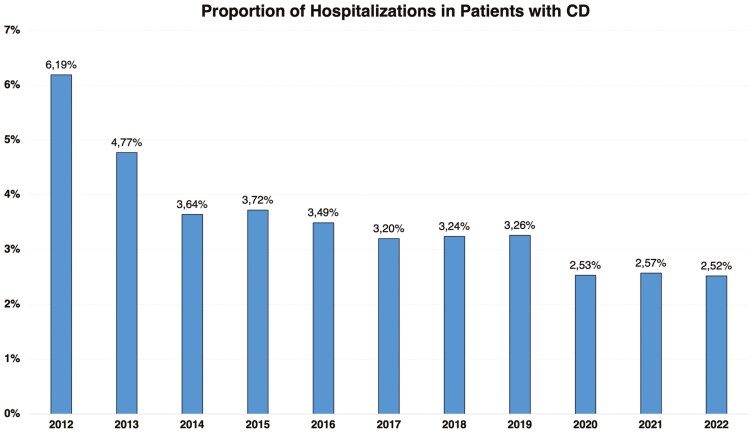
Proportion of hospitalizations and the number of patients with Crohn’s disease in Brazil’s public healthcare system from 2012 to 2022.

During the study period, the number of hospitalizations among patients with CD increased by 57.71%, totaling 24 414 admissions over the 11-year period. However, the proportion of hospitalizations decreased by 59.29%, from 6.19% in 2012 to 2.52% in 2022 (AAPC = –7.04%, 95% CI, –7.42 to –6.66; *P* < .001). Despite the rise in CD prevalence and the increased dispensing of medications during this period, hospitalizations did not grow at the same rate as the patient population. These data are presented in Table 1 and Figure 2, both in absolute and proportional terms.

**Table 1.** Number of cases and all-cause hospitalizations in patients diagnosed with Crohn’s Disease in Brazil’s public healthcare system from 2012 to 2022.

Year	CD cases (*n*)	Hospitalizations (*n*)	Hospitalizations/CD cases
2012	27 551	1705	6.19%
2013	36 410	1737	4.77%
2014	45 822	1666	3.64%
2015	54 373	2020	3.72%
2016	62 130	2167	3.49%
2017	69 977	2238	3.20%
2018	77 562	2510	3.24%
2019	85 349	2785	3.26%
2020	91 929	2330	2.53%
2021	99 816	2567	2.57%
2022	106 917	2689	2.52%

**Figure 3.**
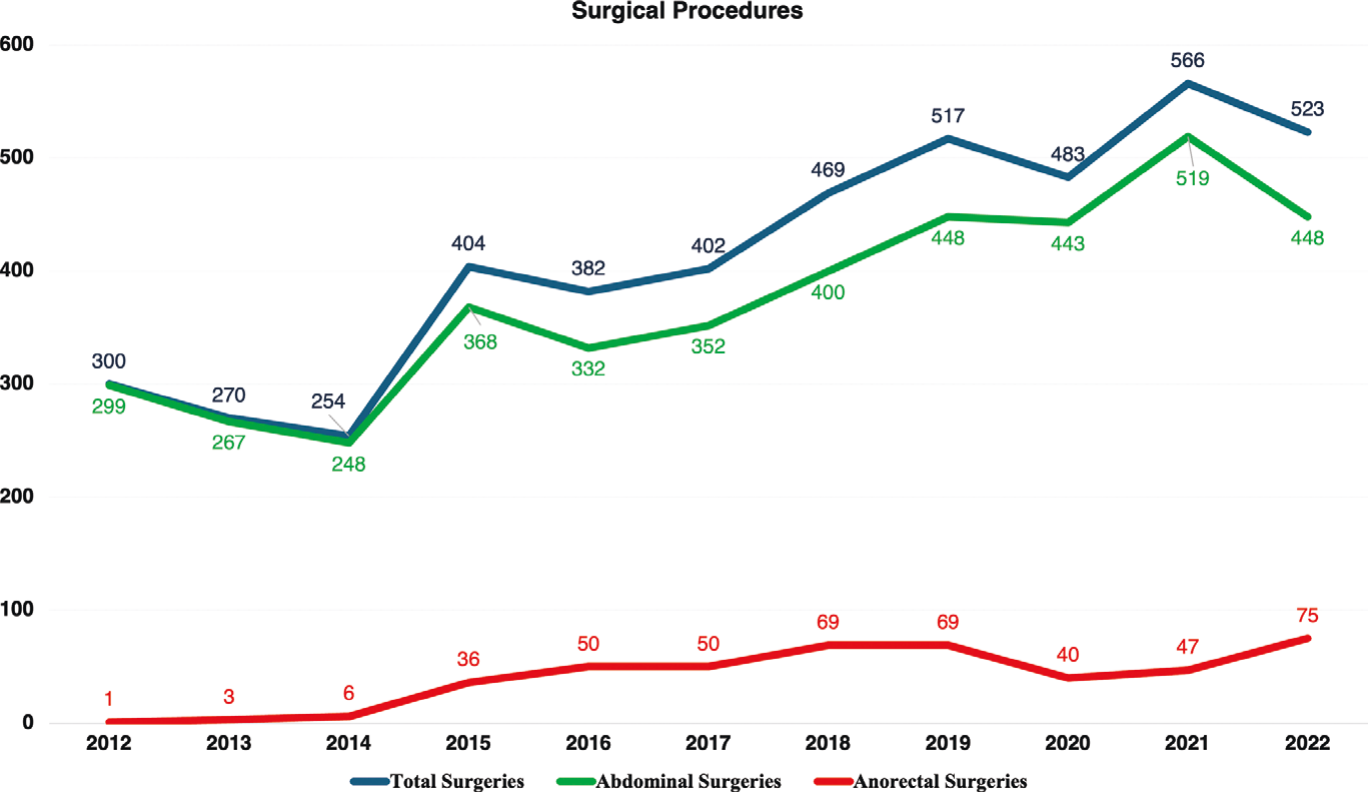
Data on abdominal and anorectal surgeries in patients diagnosed with Crohn’s disease in Brazil’s Unified Health System from 2012 to 2022.

**Figure 4.**
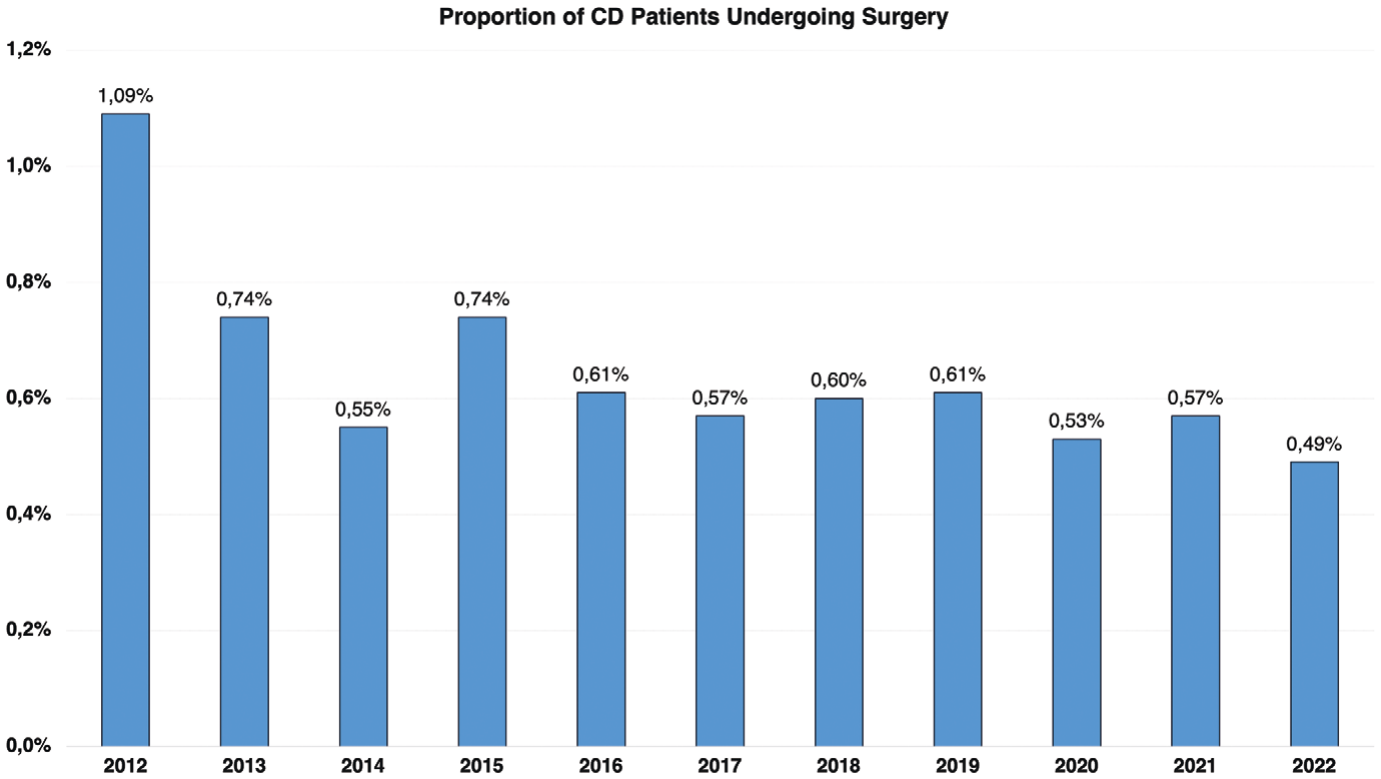
Proportion of surgeries relative to the number of Crohn’s disease cases in Brazil’s public healthcare system from 2012 to 2022.

In 2012, a total of 300 CD-related surgical procedures were recorded, of which 299 were abdominal and one was anorectal. By 2022, this number had risen to 523 procedures, with 448 abdominal and 75 anorectal surgeries, as illustrated in Figure 3 and Tables 2–4. Over the 11-year period, 4570 surgical procedures were identified, while the proportion of surgeries relative to CD cases decreased by 55.08%, from 1.09% in 2012 to 0.49% in 2022 (AAPC = –5.73%, 95% CI, –6.68 to –4.77; *P* = .001), as shown in Figure 4.

**Table 2.** Number of total surgeries, abdominal surgeries, and anorectal surgeries in patients diagnosed with Crohn’s disease in Brazil’s public healthcare system from 2012 to 2022.

Year	Total surgeries (*n*)	Abdominal surgeries (*n*)	Anorectal surgeries (*n*)
2012	300	299	1
2013	270	267	3
2014	254	248	6
2015	404	368	36
2016	382	332	50
2017	402	352	50
2018	469	400	69
2019	517	448	69
2020	483	443	40
2021	566	519	47
2022	523	448	75

**Table 3.** Number of abdominal surgeries in patients diagnosed with Crohn’s disease in Brazil’s public healthcare system from 2012 to 2022.

Abdominal surgeries	2012	2013	2014	2015	2016	2017	2018	2019	2020	2021	2022
**Enterectomy**	172	149	135	174	153	163	178	199	179	203	182
**Enterotomy and/or enterorrhaphy**	41	44	27	46	33	37	30	43	50	55	42
**Partial colectomy**	3	7	3	28	31	32	48	62	59	64	60
**Jejunostomy/ileostomy**	17	16	13	25	25	35	50	39	49	54	53
**Enteroanastomosis**	23	14	20	27	22	21	29	34	28	56	39
**Enterostomy closure**	20	21	26	36	26	30	30	30	20	30	22
**Colostomy**	6	5	5	9	13	11	14	17	22	24	26
**Colonic fistula closure**	13	4	14	7	11	4	4	2	2	7	3
**Total colectomy**	0	0	0	6	5	9	9	7	10	9	13
**Abdominal rectosigmoidectomy**	1	1	0	3	6	2	4	10	15	10	3
**Abdominal colorraphy**	2	6	5	4	2	4	3	1	4	1	1
**Complete abdominoperineal resection of rectum**	0	0	0	0	1	2	1	2	2	3	3
**Laparoscopic colectomy**	0	0	0	2	1	2	0	2	2	2	1
**Abdominoperineal rectosigmoidectomy**	1	0	0	1	3	0	0	0	1	1	0

**Table 4.** Number of anorectal surgeries in patients diagnosed with Crohn’s disease in Brazil’s public healthcare system from 2012 to 2022.

Anorectal surgeries	2012	2013	2014	2015	2016	2017	2018	2019	2020	2021	2022
**Anal fistulectomy and/or fistulotomy**	0	0	1	26	33	33	45	56	28	27	53
**Digital and/or instrumental dilation of the anus and/or rectum**	1	3	5	5	7	5	7	6	5	9	8
**Excision of anorectal lesion and/or tumor**	0	0	0	3	4	7	12	0	2	9	9
**Drainage of anorectal abscess**	0	0	0	1	5	5	4	7	3	1	5
**Drainage of ischiorectal abscess**	0	0	0	1	1	0	1	0	2	1	0

## Discussion

Our study demonstrated that despite the cumulative increase in the prevalence of CD cases and the increased dispensing of biologics, there was a reduction in hospitalizations and surgical procedures in Brazil’s public healthcare system. With the global rise in the number of IBD patients and the growing availability of biological medications, a significant proportion of CD patients undergoing admissions, whether related to surgical procedures or not, had already been exposed to this class of drugs.^18^ Despite significant advances in medical therapy for CD, surgery is still necessary for many patients and remains an important tool in treatment algorithms.^18^

As of December 2022, only 24.68% of the Brazilian population had some type of health insurance, meaning that more than two-thirds of the population relied exclusively on the public healthcare system. According to the National Supplementary Health Agency, there were 50 119 499 people covered by private health insurance companies, considering the estimated Brazilian population in 2022.^19^ These figures underscore the critical importance of the public healthcare system in serving the majority of Brazilian citizens.

The low percentage of patients receiving effective therapies, such as anti-tumor necrosis factor (TNF) or AZA, for inflammatory bowel diseases (IBDs) in Latin America reflects significant challenges, including drug licensing, high costs, and physician experience. Many countries face slow regulatory processes, delaying the availability of new advanced therapies.^20^ Additionally, the high costs of these medications limit access, especially in health systems with budget constraints. The lack of specialized training and adequate infrastructure also contributes to the preference for more traditional treatments, such as 5-ASA or steroids, which are more accessible and familiar to healthcare professionals, mostly community physicians.^21^

Insufficient healthcare infrastructure and limited awareness of IBD among the population and healthcare professionals lead to late or incorrect diagnoses, affecting the use of advanced therapies in Latin American countries.^20^ To improve the management of IBD in the region, it is crucial to develop policies that expand access to medications, increase medical training, and strengthen healthcare infrastructure.^21^ These measures could optimize health outcomes for IBD patients, promoting greater penetration of effective therapies in Latin America.

Over the 11 years analyzed (2012-2022), a total of 24 414 hospital admissions were recorded. In a 2021 study, Palacio et al. analyzed data from 2005 to 2015 and identified 20 816 hospitalizations during that period, representing a 17.28% increase when comparing the 2005-2015 period with 2012-2022.^22^ Both studies used the same data source, which explains the similarity in results. During the study period, a total of 4570 surgical procedures for CD were identified. Our data indicate an overall increase in the absolute number of surgical procedures and hospitalizations over the years. However, when examining the proportions relative to the total number of cases, there was a notable reduction. The proportion of hospitalizations decreased by 59.29%, from 6.19% in 2012 to 2.52% in 2022. Similarly, the proportion of surgeries declined by 55.08%, from 1.09% in 2012 to 0.49% in 2022. These findings suggest a shift toward more effective management strategies that may reduce the need for surgical interventions and hospital stays. These findings align with other studies that have identified decreased surgeries and hospitalizations in different countries during the biologic era.^22,23^ On the other hand, the proportion of surgeries in our study was significantly lower than that identified by Tsai et al. in a 2021 meta-analysis.^4^ Their study of 13 185 CD patients between 1955 and 2015 showed a cumulative risk of abdominal surgery of 18.7% at 1 year, 28% at 5 years, and 39.5% at 10 years. For studies from the 21st century, the cumulative risk was 12.3% at 1 year, 18% at 5 years, and 26.2% at 10 years.^4^

We believe that the number of hospital admissions and surgical procedures in our study might be significantly underestimated. Different doctors often complete ICD codes in hospitalizations. CD patients may have been admitted under other codes (such as abdominal pain or acute abdomen) or had procedure codes different from those defined in the study methodology (eg, exploratory laparotomy or abscess drainage). The same can be true for anorectal procedures, where ICD codes for fissures or fistulas could replace specific CD codes, leading to a lower capture of procedures in the platform.

Using a similar methodology, a study presented as an abstract by Magro et al. at the 2021 ECCO (European Crohn’s and Colitis Organization) congress demonstrated that the number of surgeries remained stable despite the increasing use of biologics in Brazil.^24^ Similarly, Lazarev et al. found comparable data at the University of Pittsburgh (United States), highlighting the heterogeneity in the available literature on this topic.^25^ This variability may be related to the epidemiological stage of each region. In regions where incidence has stabilized, the number of surgical procedures and hospitalizations may follow this trend. However, in developing regions with rising incidence, a concomitant increase in surgeries and hospitalizations may be observed in absolute numbers. More detailed studies are needed to evaluate whether this can be reflected in the total proportion of patients.

This study observed an increase in the absolute number of patients receiving AZA, IFX, and ADA, aligning with the rising prevalence of the disease in Brazil. However, when examining the proportion relative to the total number of cases, a significant reduction was noted in the use of AZA, which decreased by 57.28%, from 44.79% in 2012 to 19.13% in 2022. The use of IFX remained stable, shifting only slightly, from 13.83% in 2012 to 12.51% in 2022, while ADA usage experienced a modest decline of 11.75%, from 11.65% in 2012 to 10.29% in 2022. The utilization of AZA stabilized starting in 2016, whereas the trend toward increased use of biologics continued to rise. These data reflect a shift toward more effective therapies, such as anti-TNF agents, over monotherapy with immunosuppressants in the management of CD, in line with global trends and strategic studies.^26,27^

It is important to note that there was no proportional increase in the use of IFX and ADA during the study period, only in absolute numbers. Therefore, the potential cause-effect relationship between biologics and the reduction in hospitalizations and surgeries remains uncertain. A detailed causality analysis was not conducted; more studies on this topic are needed in Brazil.

Although most international studies show decreased surgery rates over time, emergency colectomy rates appear unchanged. So far, no definitive effect of reduced surgical procedures due to newer medications has been established. Whether surgery rates have decreased or been delayed with more extended follow-up observations remains debatable.^28^ Our study did not allow for an analysis of whether the surgical procedures were elective or performed in an emergency setting. As seen in other studies, this limitation hinders understanding current trends toward reducing emergency surgeries and increasing elective operations.^6^

In the present study, no important trend of reduction in drug dispensing, hospitalizations, or surgical procedures during the COVID-19 pandemic could be captured. A more detailed evaluation by month, during the pandemic period, could help to clarify this issue and warrants further research in our country.

Our study has several limitations that must be addressed for better data clarification. The inherent characteristics of this type of study, which directly analyzes electronic data from the national database, do not include specific information on disease phenotypes, longitudinal medical follow-up, postoperative complications, or comorbidities. It was also not possible to determine whether AZA was used as monotherapy or in combination with the available anti-TNF agents. Despite the availability of other types of advanced therapies in the private healthcare system, vedolizumab, ustekinumab, risankizumab, and upadacitinib are still not reimbursed in the public healthcare system. Thus, data on the use of these medications are lacking. The low number of surgical procedures suggests that the data may be underestimated, particularly in the early years of the study. Several factors may have contributed to this, such as poor completion of admission documents, with surgical findings like abscesses, perforations, and fistulas recorded as the cause of surgery without using specific CD ICD codes. Non-specialist physicians may also have entered procedure codes in the specific listing, which could have further underestimated the numbers. The lack of information on the type of surgical admission (emergency or elective) also deserves attention.

Despite these limitations, our study is one of the first population-based studies to evaluate temporal trends in the number of surgeries for CD in Latin America at a population-based level, highlighting the magnitude of the problem over a long period. It serves as a reference for future studies. Improving the completion of hospitalization and surgical forms is recommended to provide more accurate data and higher quality studies, leading to improved public policies for managing CD.

In summary, this large national population-based study demonstrated a significant increase in CD prevalence over the 11 years analyzed. A reduction in AZA use proportion, IFX stability, and a slight reduction in ADA dispensing were observed. A significant decrease in hospitalizations and surgical procedures was identified during this period. However, a clear correlation between the use of biologics and these outcomes could not be established.

Longitudinal studies conducted in reference centers for IBD management in Brazil, with large patient volumes, are necessary to confirm the temporal trends observed in these outcomes. In the context of increasing IBD prevalence, this information may help plan public policies and resource allocation for CD management in the coming years.

## Data Availability

The data that support the findings of this study are publicly available from the National Public Healthcare Department of Informatics of Brazil (DATASUS).
